# An Unusual Cause of Recurrent Syncope: Sinus of Valsalva Aneurysm

**DOI:** 10.7759/cureus.17707

**Published:** 2021-09-04

**Authors:** Muhammad Izzad Johari, Mohd Adli Haji Deraman, Mohd Sapawi Mohamed, Aimaduddin Bin Mat Daud

**Affiliations:** 1 Cardiology, Hospital Sultanah Nur Zahirah, Terengganu, MYS; 2 Cardiology, Hospital Raja Perempuan Zainab II, Kelantan, MYS

**Keywords:** sova, sinus of valsalva aneurysm, recurrent syncope, cardiac computed tomography angiography, transthoracic echocardiogram.

## Abstract

Sinus of Valsalva aneurysm (SOVA) is a rare anomaly, commonly caused by the congenital absence of elastic and muscular tissue of the sinus aortic wall. This condition often becomes apparent at the time of rupture. The most recently reported case of syncope in unruptured SOVA was due to Tachy-Brady arrhythmia involving the conduction system. This type of aneurysm also presents with signs and symptoms of ventricular outflow tract obstruction, mimicking ventricular tumor. We report a case of recurrent syncope during exertion resulting from SOVA involving the right coronary cusp, without additional symptoms such as angina, malignant arrhythmias, or infection. The mechanisms remain unknown but may be either the consequence of transient left ventricular outflow tract obstruction or cardiac arrhythmia causing syncope during exertion. This case report presents an unusual cause of syncope and demonstrates a correlation between echocardiography and CT angiography images.

## Introduction

Sinus of Valsalva aneurysm (SOVA) results from weakness of the elastic lamina that occurs at the junction of the annulus fibrosus and aortic media and can be congenital or acquired. As a rare anomaly, SOVA presents in an estimated 0.09% of the general population with a median age of 45 years old [1] and accounts for 0.1-3.5% of all congenital cardiac defects [2]. In most cases, SOVA is discovered as an incidental finding during echocardiography or other cardiac imaging. Ehlers-Danlos syndrome, Marfan syndrome, and connective tissue disease [3] are commonly associated with congenital SOVA. Around 0.5-2% of patients with a bicuspid aortic valve are likely to develop SOVA. Acquired SOVA is caused by mechanisms that lead to the weakening of elastic tissue, including infections such as syphilis, tuberculosis, and bacterial endocarditis [4].

Unruptured SOVAs are asymptomatic in most cases or may present with nonspecific symptoms and signs such as dyspnea, heart murmur palpitation, and angina. The three most common complications of SOVAs are severe aortic insufficiency, obstruction of the ventricular outflow tract or valvular orifice, and conduction disturbance [5]. Involvement of the cardiac conduction system can lead to cardiac arrhythmias, such as atrial fibrillation, Tachy-Brady arrhythmia, or complete heart block [2]. Aortic regurgitation associated with SOVA can affect up to 30-50% of patients in both ruptured and unruptured SOVAs. In addition to SOVA repair, subsequent aortic valve replacement usually is required [2].

Although echocardiography is a standard cardiac imaging modality to assess SOVA [6], cardiac CT angiography has been also widely used. This type of angiography can rule out coronary artery disease, obviating the need for an invasive coronary angiogram, which is a high-risk procedure because of the difficulty in engaging the coronary artery, a vessel prone to injury. Surgical intervention is the treatment of choice in patients with ruptured SOVA or SOVA associated with other structural heart defects, as well as in patients with symptomatic unruptured SOVA, although no SOVA-specific treatment guidelines have been established to date. For unruptured and asymptomatic SOVA, the benefit of surgical therapy is unknown.

## Case presentation

A 43-year-old man with underlying hypertension presented for evaluation due to a 1.5-year history of recurrent syncopal episodes preceded by palpitations and hyperhidrosis and it was precipitated by exertion such as running and lifting heavy objects. He had no history of seizures and he denied pre-syncopal angina, shortness of breath, or headache. He was previously healthy with no significant comorbidities. He works as a truck driver and is married with two children. He has five siblings, none of whom have similar illnesses or congenital heart disease.

**Figure 1:**
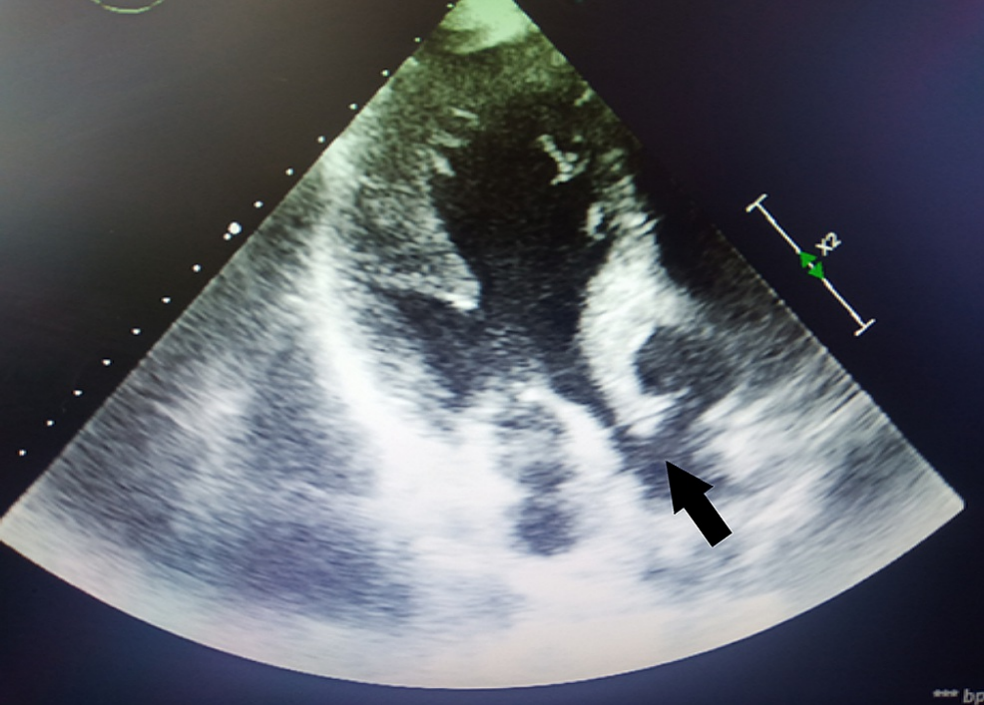
Transthoracic echocardiography. The parasternal long view shows the aneurysm sac with a compression effect to the left ventricular outflow tract.

Clinical examination on admission showed normal blood pressure with a regular heart rate. No postural hypotension was noted. Heart sounds were significant for an end-diastolic murmur, and no clinical evidence of cardiac failure was noted. Psychiatric assessment showed no specific pathology. Echocardiography showed normal sinus rhythm with right bundle branch block. Holter monitoring demonstrated no significant abnormalities. On transthoracic echocardiography to evaluate the end-diastolic murmur, an unruptured right SOVA was incidentally discovered (Figure 1).

**Figure 2:**
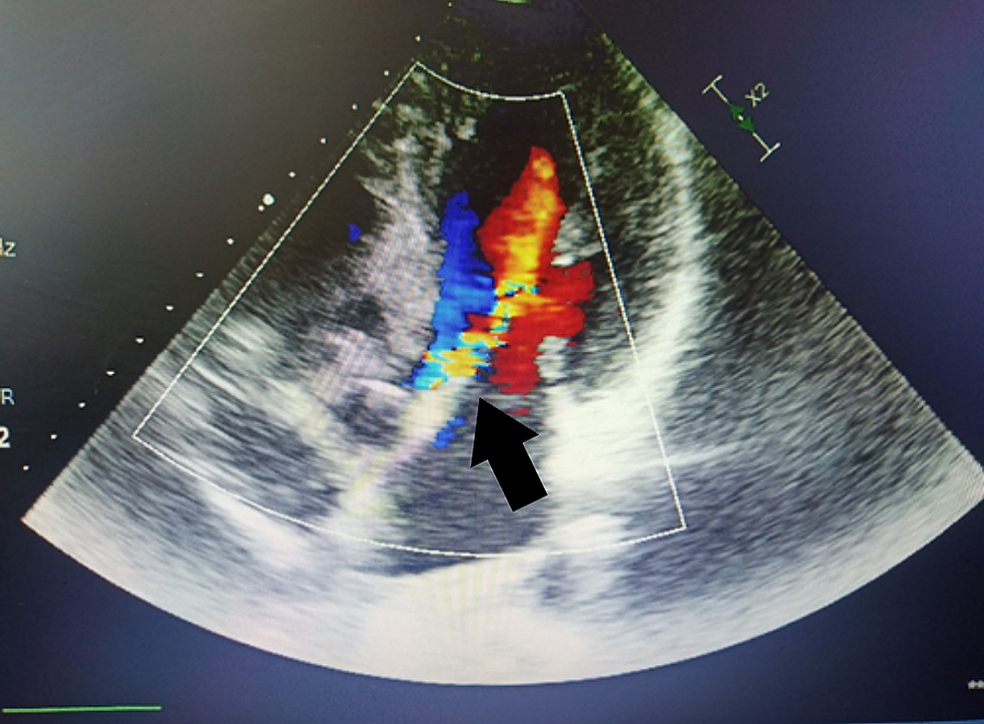
Transthoracic echocardiography. The color Doppler five-chamber view shows the finding of aortic valve insufficiency.

The patient also had moderate aortic insufficiency, in addition to mild mitral insufficiency (Figure 2).

**Figure 3:**
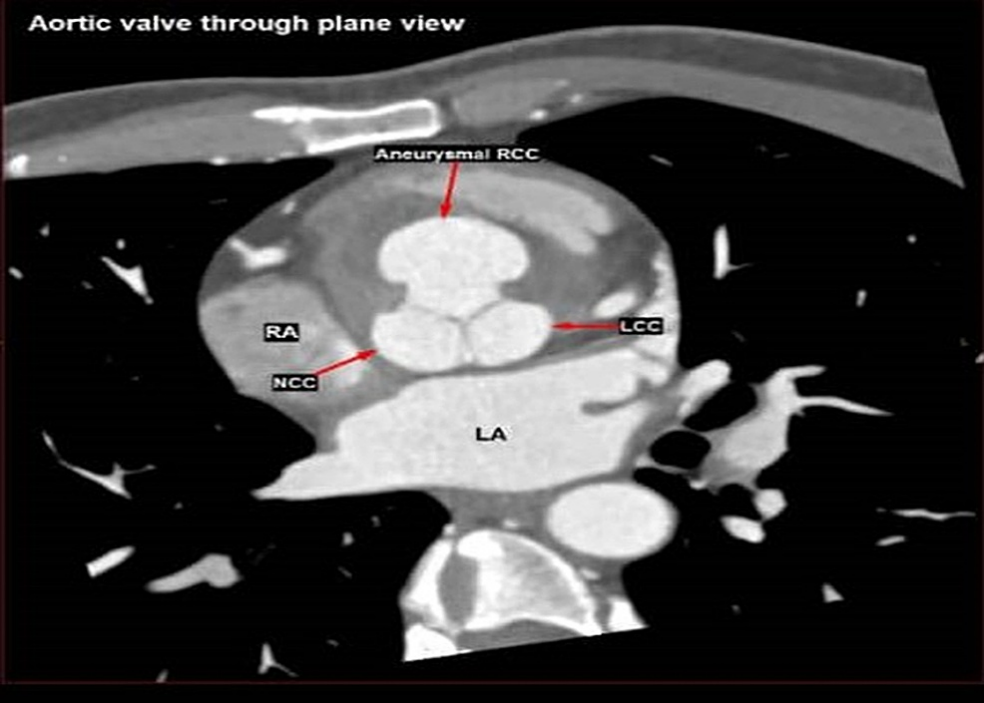
Cardiac CT scan shows the aneurysm sac in correlation to aortic valve cusps. LA: left atrium; RCC: right coronary cusp; LCC: left coronary cusp; NCC: noncoronary cusp

Urgent cardiac CT (coronary angiography and aortography) confirmed the presence of SOVA. A wide-neck saccular aneurysm originating from the right coronary cusp (Figure 3) was seen, immediately anteroinferior to the origin of the right coronary artery. The aneurysm sac measured 2.6 cm x 3.0 cm x 3.1 cm (anteroposterior x W x craniocaudal views).

**Figure 4:**
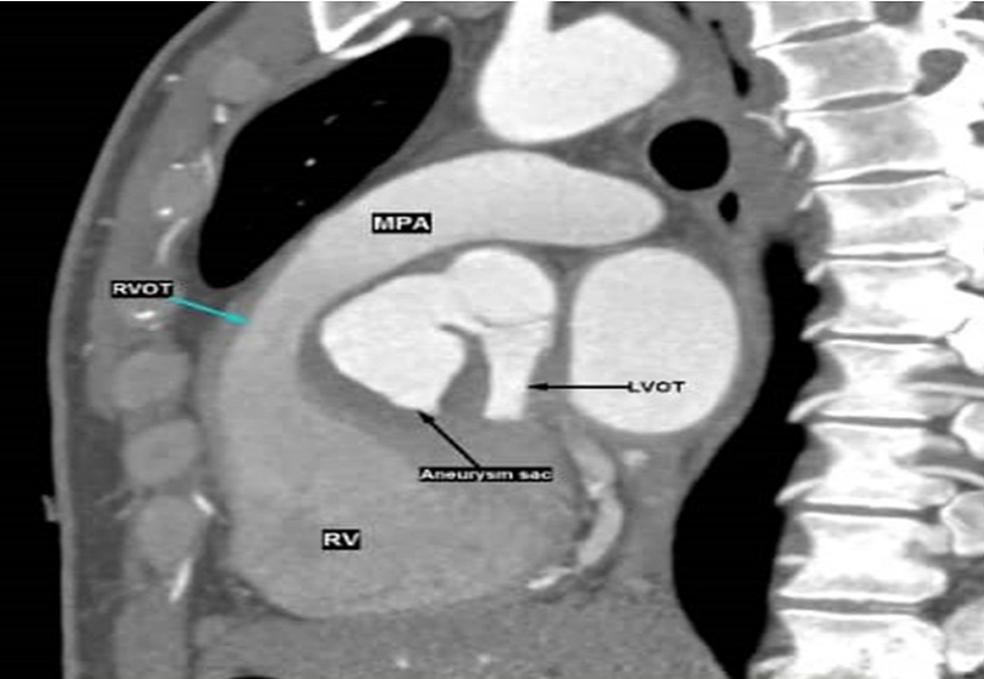
Cardiac CT scan shows the aneurysm sac with the compression effect to the right ventricular outflow tract. LVOT: left ventricular outflow tract; RV: right ventricle

The neck of the aneurysm measured 1.5 cm x 2.0 cm. The aneurysm sac extended inferiorly into the interventricular septum. A smooth peripheral non-enhancing hypodensity was visualized within the aneurysmal sac, likely representing a thrombus. Anteriorly, the sac caused the narrowing of the right ventricular outflow tract (RVOT), measuring 1.1 cm (Figure 4).

**Figure 5:**
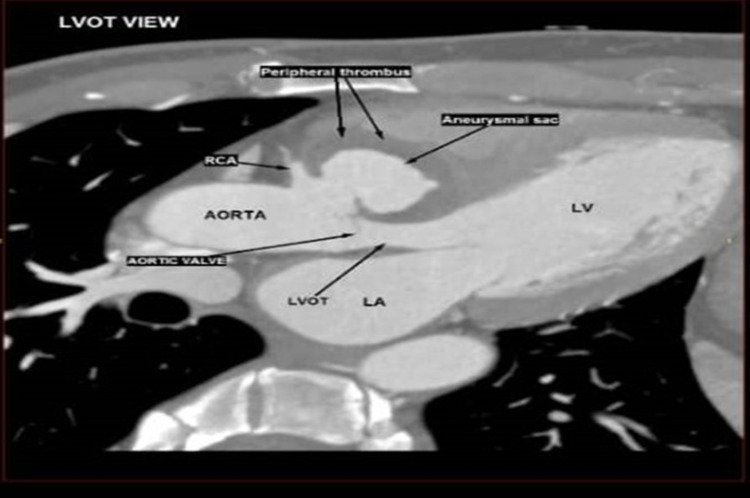
Cardiac CT showing the aneurysm sac with compression effect to the left ventricular outflow tract. RCA: right coronary artery; LV: left ventricle; LVOT: left ventricular outflow tract

Posteriorly, it caused a narrowing of the left ventricular outflow tract (LVOT), measuring 0.9 cm (Figure 5). The sac also appears to be in close proximity with the septal leaflet of the tricuspid valve. The presence of fat stranding surrounding the aortic root was observed. No contrast extravasation or perianeurysmal collection was seen. The thoracic aorta was normal in size. No evidence of aneurysm, coarctation, or dissection was seen. The aortic valve was tri-leaflet. There was no valvular thickening, calcification, or mass/thrombus.

**Figure 6:**
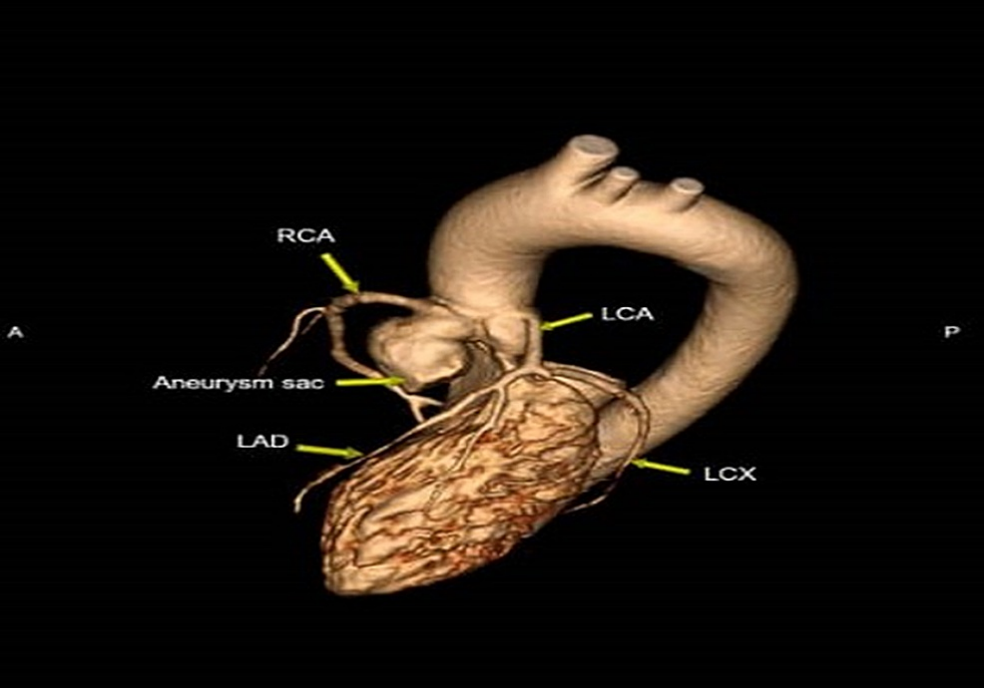
Cardiac angiography scan shows the aneurysm sac in correlation to the coronary arteries and aorta. RCA: right coronary artery; LCA: left coronary artery; LAD: left anterior descending; LCX: left circumflex

Coronary arteries were normal with calcium score of 0 (Figure 6). The extra-coronary cardiovascular findings showed normal systemic and pulmonary venous return, no obvious resting myocardial perfusion defect, intact appearance of the intra-atrial septum and no thrombus or mass in the cardiac chamber.

The patient was referred to the surgical team for further intervention to manage his symptomatic unruptured saccular aneurysm of the right sinus of Valsalva. However, the patient refused further intervention and opted for conservative management.

A month after the event, the patient was asymptomatic and able to return to work, although he had not yet resumed the activities of his job that required lifting heavy objects and climbing more than three flights of stairs. Subsequent follow-up visits at our clinic were scheduled to monitor the symptoms and progression of the aneurysm, and the patient agreed to further intervention if he developed another syncopal or cardiac event.

## Discussion

SOVA is a rare congenital or acquired anomaly caused by any conditions affecting the aortic wall, such as connective tissue disease, infection, or a congenital condition. These aneurysms have been increasingly diagnosed with the widely assessable use of imaging modalities and improved imaging techniques. The common presentation of an unruptured SOVA is heart failure due to aortic insufficiency, angina, tachyarrhythmias, and infection. Our patient presented with recurrent syncope that occurred during exertion. Syncope associated with unruptured SOVA is a recognized symptom of this condition. In previously reported cases of SOVA, symptoms were due to Tachy-Brady arrhythmia and transient ventricular outflow obstruction. Sohal et al. reported a case of syncope secondary to hypotension resulting from reduced left atrial filling as a consequence of transient RVOT obstruction [7]. Furthermore, SOVAs can also be complicated with aortic insufficiency, with an incidence in up to 50% of patients with SOVA; likewise, moderate aortic insufficiency was noted in our patient [2]. Based on our patient presentation, the recurrent syncope was likely caused by transient ventricular outflow obstruction as visualized on the CT angiography showing the narrowing of both ventricular outflows due to the extension of the aneurysm, which may have led to transient ventricular outflow obstruction precipitated by exertion.

Transthoracic and transesophageal echocardiography is a fast, non-invasive way to assess and provide information on characteristics of aneurysmal dilatation, cardiac chamber involvement, fistulous tracts, and valvular assessment, as well as any associated cardiac anomalies [6]. Echocardiographic evaluation combined with color Doppler studies is used to evaluate ruptured SOVAs by demonstrating the presence of continuous flow in systole and diastole. In this patient, transthoracic echocardiography was performed to assess the valvular function, also yielding the incidental finding of an unruptured right SOVA. Other supplementary or confirmatory imaging techniques used are magnetic resonance, contrast aortography, and CT. With the current advancement of cardiac CT as imaging technology, it is now possible to capture images during less than a single cardiac cycle using electrocardiography-gated technology and wide-range rotation [8]. For this patient, CT angiography of the thoracic aorta with retrospective electrocardiography gating was performed, and the findings confirmed a right wide-neck saccular aneurysm of right sinus of Valsalva, complicated with RVOT and LVOT narrowing and possible abutment on the septal leaflet of the tricuspid valve, with no evidence of rupture or collection.

Surgical intervention is recommended in ruptured SOVA and SOVAs with intracardiac abnormalities such as severe aortic insufficiency, ventricular defect, or other associations with another congenital heart disease. Alternative treatment includes utilizing percutaneous closure repair using Amplatzer™ Duct Occluder (Abbott, Chicago, IL, US), demonstrating its effectiveness and feasibility. In patients with unruptured and asymptomatic SOVA, the role of surgical intervention remains unclear [9]. The decision to repair is based on concurrent factors such as Tachy-Brady arrhythmia, outflow tract obstruction, infection, and symptomatic presentation. The first successful surgical intervention for a SOVA was in 1957 and, since then, multiple approaches and techniques have been developed. Based on a low mortality rate (1.9%-3.6%) and excellent survival rate (up to 90% at 15 years), surgical repairs are recommended for SOVA [10,11]. In patients with an unruptured but symptomatic or enlarging aneurysm, surgical repair is considered, although specific recommendations and guidelines for repair have yet to be established. For our patient, we advised and recommended that he pursue surgical intervention due to his recurrent syncope related to SOVA; however, the patient opted for conservative management and is now scheduled for regular follow-up at our hospital.

## Conclusions

SOVA is a cardiac anomaly that can be congenital or acquired. Transthoracic color Doppler echocardiography is the initial imaging technique and further imaging with cardiac MRI and CT as the confirmative diagnostic tools. This entity is increasingly diagnosed as a result of advances in cardiac imaging technology. Recommended indications for surgical intervention are ruptured, symptomatic unruptured, and unruptured SOVAs associated with concurrent factors, such as outflow tract obstruction, arrhythmia, or infection. This case report presented a patient with symptomatic unruptured SOVA and recurrent syncope. The indication for surgical intervention was prompted by his symptoms of recurrent syncope to prevent another syncopal episode and further complications. With the increasing prevalence of SOVAs detected as an incidental finding on cardiac imaging, a randomized controlled trial of intervention for SOVA versus other treatment options will be beneficial to optimize the management of patients with this rare cardiac anomaly, especially in cases of an unruptured SOVA, to prevent future complications.
